# Traumatic common iliac artery occlusion mimicking acute sciatica: a case report

**DOI:** 10.1093/jscr/rjag506

**Published:** 2026-06-22

**Authors:** Guan-Yu Chen, Te-Chang Wu, Jinn-Rung Kuo

**Affiliations:** Department of Neurosurgery, Chi-Mei Medical Center, Chung Hwa Road, Yung Kang District, 71049 Tainan, Taiwan; Department of Medical Imaging, Chi-Mei Medical Center, Chung Hwa Road, Yung Kang District 71049, Tainan, Taiwan; Department of Neurosurgery, Chi-Mei Medical Center, Chung Hwa Road, Yung Kang District, 71049 Tainan, Taiwan; School of Medicine, College of Medicine, National Sun Yat-sen University, No. 70, Lianhai Rd., Gushan Dist., Kaohsiung City 804201, Taiwan

**Keywords:** common iliac artery occlusion, blunt trauma, sciatica mimic, vascular injury, endovascular treatment

## Abstract

Lower extremity numbness, pain, and weakness after trauma are often attributed to neurological causes such as lumbar disc herniation or spinal injury. However, vascular injuries may present with similar symptoms and lead to diagnostic confusion. We report a 61-year-old woman who developed progressive numbness and weakness of the right lower extremity after a traffic-related fall onto the right hip. Lumbar magnetic resonance imaging showed L5 endplate fracture and degenerative spinal changes, initially suggesting a neurogenic cause. However, physical examination revealed pallor, coldness, and absent distal pulses in the affected limb, raising suspicion of arterial occlusion. Computed tomography angiography confirmed complete occlusion of the right common iliac artery. The patient underwent endovascular angioplasty with stent placement, which restored arterial flow and resulted in rapid symptom resolution. This case highlights an important diagnostic pitfall and emphasizes the importance of careful pulse examination when post-traumatic neurological symptoms are accompanied by limb ischemia.

## Introduction

Blunt pelvic trauma may occasionally result in vascular injury; however, isolated iliac arterial occlusion without an associated pelvic fracture is rare [1, 2]. Such injuries can present with symptoms that resemble neurological disorders, including lumbar disc herniation or spinal stenosis, creating a diagnostic challenge and increasing the risk of delayed treatment [3]. Previous reports of iliac or femoral arterial occlusion after blunt trauma have highlighted the potential for vascular lesions to masquerade as neurological conditions. Accurate distinction between neurogenic and vascular causes of lower extremity symptoms is therefore essential. Here, we report a rare case of right common iliac artery occlusion after blunt trauma to the right hip that initially presented with acute sciatica-like symptoms.

## Case presentation

A 61-year-old woman with no significant medical history presented to the emergency department with progressive numbness and weakness of the right lower extremity after a traffic-related fall onto the right buttock, followed by localized pain. Approximately 30 min later, she developed progressive numbness radiating from the buttock to the foot, accompanied by weakness of the affected limb.

On arrival, she was alert with a Glasgow Coma Scale score of 15. Her blood pressure was 120/77 mmHg and heart rate was 102 beats/min. Neurological examination revealed muscle strength of 1–2/5 in the right foot on the Medical Research Council scale. The straight-leg-raising test was positive at 60° on the right and 90° on the left. There was no urinary incontinence or anal sphincter dysfunction.

Lumbar spine magnetic resonance imaging demonstrated an L5 superior endplate fracture, along with herniated intervertebral discs and spinal canal stenosis at L2–3 and L4–5, as well as grade I spondylolisthesis (Fig. 1a and b). However, these imaging findings did not fully explain the severity and distribution of the patient’s symptoms.

**Figure 1:**
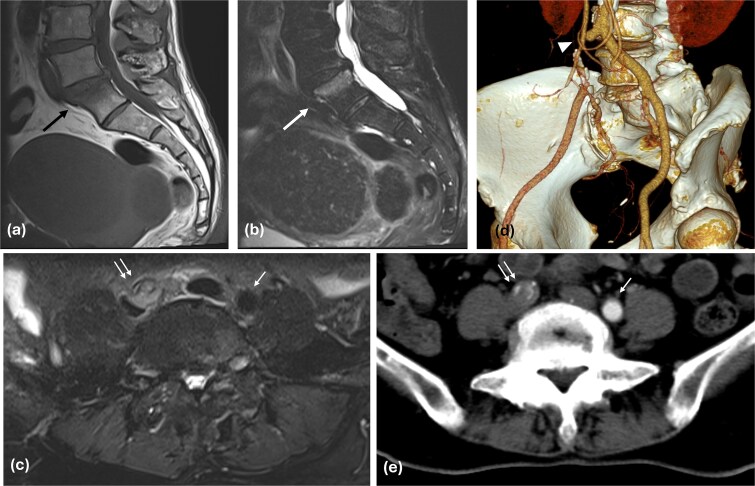
Imaging findings. (a) Sagittal spin-echo (SE) T1-weighted and (b) sagittal fast spin-echo (FSE) T2-weighted lumbar spine images showing a fracture line through the superior endplate of L5 (long arrow) with focal marrow edema and L2–3 disc herniation. (c) Axial FSE T2-weighted image with fat saturation at L4–5 showing occlusion of the right common iliac artery (double arrow) with loss of flow void, compared with the patent left common iliac artery (short arrow). (d) Three-dimensional CT angiography showing occlusion of the right common iliac artery (arrowhead) anterior to the L4–5 disc level with distal collateral reconstitution. (e) Contrast-enhanced CT at L4–5 showing near-complete occlusion of the right common iliac artery with a small residual lumen.

Further bedside examination revealed that the right lower extremity was pale and cold. The femoral pulse was weak, whereas the popliteal and dorsalis pedis pulses were absent. These findings raised suspicion of acute arterial occlusion. Computed tomography (CT) angiography confirmed complete occlusion of the right common iliac artery with reduced distal perfusion (Fig. 1c–e).

The patient was treated with enoxaparin and aspirin and subsequently underwent endovascular angioplasty with stent placement (Fig. 2), which successfully restored arterial flow.

**Figure 2:**
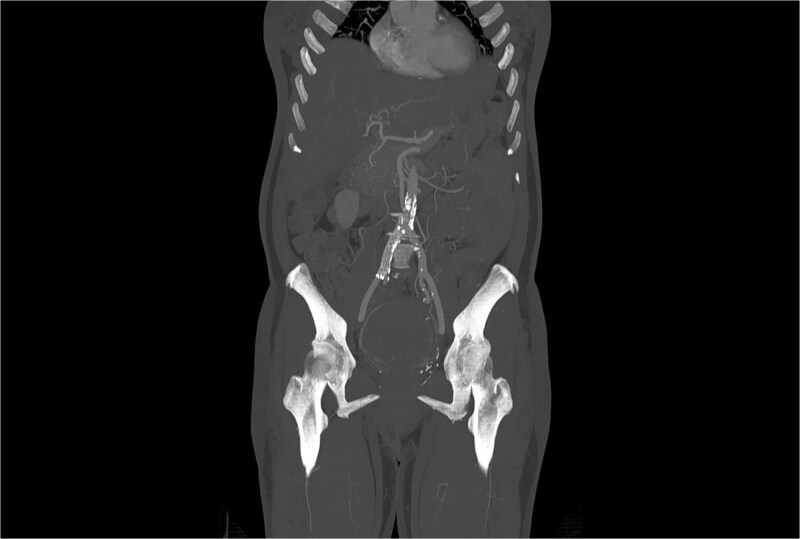
Endovascular angioplasty with stent placement successfully restoring patency of the right common iliac artery. CT angiography, coronal view. The right common iliac artery is patent at the L4–5 level. A previously implanted stent is seen extending from the L4 to S1 level of the right common iliac artery.

Following revascularization, her symptoms rapidly improved. The classic signs of acute limb ischemia—pain, paresthesia, pallor, pulselessness, paralysis, and poikilothermia—resolved. She was discharged on postoperative day 5 with dual antiplatelet therapy.

## Discussion

Vascular injury after blunt pelvic trauma is uncommon and is typically associated with high-energy mechanisms or pelvic fractures. In contrast, iliac artery occlusion after blunt trauma without associated pelvic fracture, despite concomitant spinal injury, remains particularly rare [1, 2]. Previous reports have described iliac or femoral arterial occlusion following blunt trauma without pelvic fracture, highlighting that clinically significant vascular injury may occur even after blunt trauma [4–6].

The primary diagnostic challenge in the present case was the overlap between symptoms of acute limb ischemia and lumbar radiculopathy. Pain, numbness, and weakness of the lower extremity are common manifestations of both conditions. When lumbar imaging demonstrates degenerative abnormalities such as disc herniation or spinal stenosis, clinicians may be inclined to attribute neurological symptoms to a spinal cause [3]. However, degenerative spinal findings are common in older adults and may not necessarily explain the severity or rapid progression of neurological deficits. In our patient, lumbar MRI revealed disc herniation and spondylolisthesis, which initially suggested a neurogenic origin of symptoms. Nevertheless, careful bedside examination revealed pallor, coldness, and absence of distal pulses, which are classic signs of acute limb ischemia. These physical findings prompted further vascular evaluation and ultimately led to the correct diagnosis.

The pathophysiological mechanism of traumatic iliac artery occlusion is thought to involve intimal injury caused by compression or stretching forces during blunt trauma, leading to thrombosis and subsequent arterial occlusion [1, 2, 4]. In patients with underlying atherosclerotic disease, the arterial wall may already be vulnerable to plaque disruption. Blunt trauma may therefore trigger endothelial injury, plaque rupture, and thrombosis in susceptible individuals. In the present case, diffuse atherosclerotic changes were noted in the abdominal aorta and femoral arteries, suggesting that pre-existing vascular pathology may have predisposed the patient to acute thrombosis after trauma.

Another notable finding in this case was the coexistence of an L5 vertebral fracture and occlusion of the right common iliac artery at a similar anatomical level. Although a direct causal relationship cannot be definitively established, it is possible that the vertebral fracture resulted in localized compression or stretching of the adjacent vessel, leading to intimal disruption and subsequent thrombosis. Alternatively, the traumatic force transmitted to the pelvis may have caused transient arterial deformation against the vertebral body or surrounding structures. These mechanisms have been proposed in previous reports describing blunt iliac artery injuries without pelvic fracture [4–6].

Delayed recognition of arterial occlusion may lead to serious complications, including prolonged limb ischemia, irreversible nerve injury, compartment syndrome, and even limb loss [7]. For this reason, assessment of limb perfusion and distal pulses should be an integral component of the initial evaluation in trauma patients presenting with lower extremity neurological symptoms. This is particularly important when the neurological findings appear disproportionate to spinal imaging abnormalities or when symptoms progress rapidly.

From a therapeutic perspective, endovascular intervention has become an increasingly important treatment option for traumatic iliac artery injuries. Compared with open surgical repair, endovascular angioplasty with stent placement offers the advantages of lower invasiveness, shorter operative time, and rapid restoration of arterial flow. Recent studies have reported favorable outcomes with endovascular management in selected patients with traumatic iliac artery injury [6, 8–10]. In our patient, prompt endovascular revascularization successfully restored perfusion and resulted in rapid resolution of ischemic symptoms, highlighting the effectiveness of this minimally invasive approach.

This case illustrates an important diagnostic pitfall in trauma evaluation. Iliac artery occlusion after blunt trauma may present with symptoms resembling acute lumbar radiculopathy, particularly when spinal imaging reveals degenerative abnormalities. When neurological symptoms are accompanied by signs suggestive of limb ischemia—such as pallor, coldness, or diminished pulses—clinicians should maintain a high index of suspicion for vascular injury. Early recognition and timely revascularization are essential to prevent delayed diagnosis and potentially limb-threatening complications.

## Conclusion

Isolated iliac artery occlusion after blunt trauma is rare but may present with symptoms resembling lumbar radiculopathy. This case emphasizes that careful physical examination, particularly assessment of distal pulses, remains essential when neurological findings do not match imaging results. Early recognition and prompt revascularization are crucial to prevent limb-threatening complications. Vascular injury should be considered when post-traumatic radiculopathy-like symptoms are accompanied by limb ischemic signs.
